# Peroxisome Proliferator-Activated Receptors Protect against Apoptosis via 14-3-3

**DOI:** 10.1155/2010/417646

**Published:** 2010-08-24

**Authors:** Kenneth K. Wu

**Affiliations:** Institute of Cellular and System Medicine, National Health Research Institutes, 35 Keyan Road, Zhunan, Miaoli County 350, Taiwan

## Abstract

Peroxisome proliferator-activated receptors (PPARs) were reported to prevent cells from stress-induced apoptosis and protect tissues against ischemia-reperfusion injury. The underlying transcriptional mechanism is unclear. Recent reports indicate that the antiapoptotic actions of ligand-activated PPAR*δ* and PPAR*γ* are mediated through enhanced binding of PPAR to the promoter of 14-3-3*ε* and upregulation of 14-3-3*ε* expression. We propose that ligand-activated PPAR*α* exerts its anti-apoptotic actions via the identical pathway. The PPAR to 14-3-3 transcriptional axis plays an important role in protection of cell and tissue integrity and is a target for drug discovery.

## 1. Introduction

Peroxisome proliferator-activated receptors (PPAR) are nuclear receptors that mediate diverse metabolic and cellular functions. They comprise three members: PPAR-*α*, PPAR-*γ*, and PPAR-*δ* (also known as PPAR-*β*), which have a high degree of sequence homology and share common structural characteristics (For review see [1]). In addition to their well-recognized actions on regulating lipid metabolism and glucose homeostasis, PPARs are involved in diverse functions such as cell survival, proliferation, differentiation and inflammation [2, 3]. There is an increasing evidence that all three PPAR isoforms are crucial for defending against apoptosis induced by oxidative and metabolic stresses. However, the mechanism by which ligand-activated PPARs defend against apoptosis is largely unknown. Recently, it was reported that ligand-activated PPAR*δ* and PPAR*γ* exert anti-apoptotic actions by transcriptional upregulation of 14-3-3*ε* [4]. Here, we review the reported data and propose a common anti-apoptotic mechanism.

## 2. Prostacyclin Protects Cells from Stress-Induced Apoptosis

Prostacyclin (PGI_2_) is a metabolite of arachidonic acid (AA). Its biosynthesis is requires the coordinated actions of (1) phospholipase A_2_ which liberates AA from membrane phospholipids, (2) cyclooxygenase (COX, also known as prostaglandin H synthase) which converts AA into PGH_2_, and (3) prostacyclin synthase (PGIS) which converts PGH_2_ into PGI_2_ [5]. The PGI_2_ synthetic enzymes are expressed in several cell types including vascular endothelial and smooth muscle cells, cardiac cells, renal interstitial cells, and certain cancer cells. PGI_2_ possesses multiple biological actions and plays important roles in important physiological and pathological functions. Extensive investigations have established its platelet inhibitory and vasodilatory actions and its essential function in vascular homeostasis [6–8]. The classic actions of PGI_2_ on inhibition of platelet aggregation and vasoconstriction are mediated via I-type prostaglandin (IP) membrane receptor which signals through protein kinase A pathway [9]. Recent studies have reported that PGI_2_ protects diverse cells against stress-induced apoptosis; it protects renal interstitial cells from hypertonicity-induced apoptosis, cardiomyocytes from doxorubicin-induced apoptosis and megakaryocytes from nitric oxide-(NO-) induced apoptosis. [10–12]. The published reports imply that its anti-apoptotic action is mediated via PPAR. First, synthetic PGI_2_ analogs including carbaprostacyclin (cPGI_2_) and iloprost were reported to bind PPAR*δ* and PPAR*α* [13]. Second, protection of renal interstitial cells against hypertonicity-induced apoptosis by PGI_2_ was correlated with PPAR*δ* activation [14]. Third, PPAR*δ* was reported to protect against apoptosis in keratinocytes [15], cardiomyocyte [16], islet *β* cell [17], and smooth muscle cells [18]. To ascertain that authentic PGI_2_ protects endothelial cells against apoptosis via PPAR*δ*, Liou et al. transduced human umbilical vein endothelial cells (HUVECs) with an adenoviral vector containing bicistronic COX-1 and PGIS cDNA (Ad-COPI), which expresses abundant COX-1 and PGIS and consequently produce a large quantity of PGI_2_ by shunting the arachidonate metabolism through the COX/PGIS pathway [19]. HPLC analysis reveals a marked elevation of PGI_2_ without an increase in any other prostaglandins in Ad-COPI transfected cells. Ad-COPI transfected cells are highly resistant to apoptosis induced by H_2_O_2_ [4]. Intraventricular infusion of Ad-COPI into ischemic brain significantly reduces infarct volume induced by ischemia-reperfusion (I/R) in a rat stroke model [19]. Intraventricular infusion of Ad-COPI in rats is accompanied by a 4-fold increase in PGI_2_ and a significant reduction of other prostaglandins and leukotrienes in the ipsilateral brain tissues, consistent with a metabolic shift to PGI_2_ synthesis *in vivo* [19]. Administration of Ad-COPI to rats several hours after I/R injury remains effective in reducing cerebral infarction volume [19]. These results suggest that authentic PGI_2_ production via Ad-COPI transfection suppresses apoptosis and reduces the extent of brain infarction. 

The anti-apoptotic effect of Ad-COPI in HUVECs is abrogated by cotransfection with a selective PPAR*δ* small interference RNA (siRNA) but not a control RNA. It is estimated that the authentic PGI_2_ generated by gene transfer is effective in protecting against apoptosis and I/R-induced damage at nM concentrations. In contrast, PGI_2_ analog, cPGI_2,_ inhibits H_2_O_2_-induced HUVEC apoptosis at 10–50 *μ*M. L-164051, a synthetic PPAR*δ* ligand, is as effectively as cPGI_2_ in blocking H_2_O_2_-induced apoptosis, and the anti-apoptotic effects of cPGI_2_ and L-165041 are abrogated by PPAR*δ* siRNA. Western blot analysis shows that HUVECs express abundant PPAR*δ* proteins. Ad-COPI as well as cPGI_2_ and L-165041 activates the expression of luciferase in cells transfected with a PPAR promoter-luciferase construct, consistent with expression of functional PPAR*δ* in HUVEC. These results indicate that the authentic PGI_2_ generated endogenously by gene transfer or its synthetic analogs such as cPGI_2_ protect endothelial cells against oxidant-induced cell death via PPAR*δ*.

## 3. Ligand-Activated PPAR*δ* Binds and Upregulates 14-3-3*ε* Promoter

14-3-3 is identified as a target of ligand-activated PPAR*δ* through candidate gene screening. 14-3-3 proteins function as a scaffold to regulate the activities of kinases, facilitate intracellular translocation of diverse proteins, and control apoptosis [20]. Human 14-3-3 comprises seven members, all of which are constitutively expressed in HUVECs. cPGI_2_ and L-165041 increase the expression primarily of 14-3-3*ε* proteins [4]. PPAR*δ* ligands stimulate the 14-3-3*ε* promoter activity to an extent comparable to 14-3-3 protein. 14-3-3*ε* promoter does not have TATA-box but harbors three PPAR response elements (PPRE) [4]. Deletion of the PPRE elements from the promoter construct abolishes the promoter stimulating effect of cPGI_2_ or L-165041. Analysis of PPAR*δ* binding to the PPRE region by chromatin immunoprecipitation reveals that PPAR*δ* ligands enhance binding of PPAR*δ* to the PPRE-containing fragment but not to a distal segment that does not contain PPRE motifs. Thus, ligand-activated PPAR*δ* binds directly to its binding sites on 14-3-3*ε* promoter and upregulates 14-3-3*ε* expression.

## 4. PPAR*δ*-Mediated 14-3-3*ε* Upregulation Enhances Bad Sequestration

The constitutively expressed 14-3-3*ε* proteins serve as a gatekeeper to defend against apoptosis via the mitochondrial death pathway by sequestering Bad, Bax, and Foxo [21]. However, the basal 14-3-3 levels are inadequate for controlling apoptosis when the cells are challenged with excessive stresses. The ligand-activated PPAR*δ* plays an important role in conferring the anti-apoptotic defense by upregulating 14-3-3*ε* expression. An increase of 14-3-3*ε* proteins by PGI_2_- or L-165041-activated PPAR*δ* enhances significantly Bad sequestration. Results from immunoprecipitation experiments confirm enhanced Bad binding by 14-3-3*ε* in cells treated with PPAR*δ* ligands. Analysis of subcellular localization of Bad shows reduced Bad translocation to mitochondria and a reciprocal accumulation of Bad in cytosolic fractions of cells treated with PPAR*δ* ligands compared to control. Consistent with reduced Bad translocation to mitochondria, mitochondrial membrane potential is restored and release of cytochrome C and Diablo is suppressed in H_2_O_2_-treated cells supplemented with PPAR*δ* ligands [22]. Taken together, these results indicate that 14-3-3*ε* upregulation by PPAR*δ* ligands has an important functional impact on controlling oxidant-induced apoptosis.

## 5. Nonsteroidal Anti-Inflammatory Drugs Induce Apoptosis by Suppressing PPAR*δ*/14-3-3*ε*

A number of nonsteroidal anti-inflammatory drugs (NSAIDs) induce normal and cancer cell apoptosis in a cyclooxygenase-2-(COX-2-) dependent or independent manner [23–25]. The exact mechanisms by which NSAIDs induce apoptosis are not entirely clear. One potential mechanism involves the PPAR*δ* transcriptional pathway. It was reported that PPAR*δ* in colorectal cancer cells promotes cell proliferation [26, 27] and NSAIDs induce colon cancer cell apoptosis by suppressing PPAR*δ* [28]. Results from our laboratories have shown that sulindac sulfide and indomethacin suppress PPAR*δ* expression with corresponding inhibition of 14-3-3*ε* promoter activity and protein expression [29]. Downregulation of 14-3-3*ε* is accompanied by reduced Bad sequestration by 14-3-3*ε* and increased translocation of Bad to mitochondria leading to apoptosis via the mitochondrial death pathway. NSAID-induced apoptosis is attenuated by 14-3-3*ε* overexpression. The proapoptotic effect of NSAIDs is not restricted to cancer cells. Sulindac and indomethacin induce HUVEC apoptosis by suppressing PPAR*δ*/14-3-3*ε* and thereby enhancing Bad-mediated cell death via mitochondrial damage [30]. Thus, suppression of PPAR*δ*/14-3-3*ε* transcriptional pathway represents a major mechanism by which NSAIDs induce cell death.

## 6. Conflicting Effects of PPAR*γ* Agonists on Cell Survival

PPAR*γ* agonists such as thiazolidinediones (for example, rosiglitazone, and pioglitazone) and prostaglandin D_2_ metabolites (15-deoxy-Δ^12,14^-PGJ_2_) regulate cell survival but the results are conflicting. PPAR*γ* agonists were reported to induce apoptosis in different types of cells including endothelial cells, vascular smooth muscle cells, and cancer cells [31, 32]. On the other hand, rosiglitazone was reported to protect cardiomyocytes, *β* islet cells, and neurons against apoptosis [33–35]. The reasons for the conflicting results in those reports are unclear but may be explained by use of different concentrations of PPAR*γ* agonists, different cell types, and/or PPAR*γ*-independent actions of the agonists [36]. It was reported that thiazolidinediones at concentrations that activate the PPAR*γ* transcriptional activity protect cell survival while at higher concentrations they induce apoptosis [37]. We have evaluated concentration-dependent effects of rosiglitazone on neuronal apoptosis and I/R brain damage. Rosiglitazone exerts a biphasic effect on hypoxia/reoxygenation-induced neuronal apoptosis and I/R-induced brain damage. At low *in vitro* concentrations (<5 *μ*M) and low *in vivo* doses (<50 ng) in a rat stroke model, rosiglitazone protects against neuronal apoptosis and attenuates cerebral infarct volume while at high concentrations and doses, rosiglitazone does not have any protective effect and may aggravate the hypoxia and ischemia-induced cell and tissue damage [35]. The mechanism by which thiazolidinedione and 15d-PGJ_2_ exert a biphasic concentration-dependent effect on cell and tissue protection is unclear and requires further investigations.

## 7. Rosiglitazone Protects against Ischemia/Reperfusion-Induced Cerebral Infarction via PPAR*γ*-Mediated 14-3-3*ε* Upregulation

In order to understand how PPAR-*γ* agonists reduce brain tissue damage by I/R, we have evaluated the effect of 15d-PGJ_2_ (10 pg) or rosiglitazone (50 ng) on I/R-induced infarction volume by intraventricular infusion. At the relatively low doses used, the PPAR-*γ* agonists reduced the infarct volume to a similar extent [35, 38]. Further investigations reveal that rosiglitazone is effective in reducing the infarct volume when it is infused 2 hours after I/R [35]. The protective effect of rosiglitazone is abrogated by GW9662, a PPAR*γ* antagonist as well as by PPAR*γ* siRNA. On the other hand, cerebral infarction is rescued by overexpression of PPAR*γ*. Results from those studies indicate that PPAR*γ* agonists at appropriate “therapeutic” doses protect brain tissues from I/R damage in a PPAR*γ*-dependent manner. 15d-PGJ_2_ and rosiglitazone administration is accompanied by a significant reduction of apoptotic markers in the I/R damaged brain [35, 38]. The *in vitro* cellular studies have revealed that rosiglitazone protects neurons from apoptosis induced by hypoxia/reoxygenation [35]. Taken together, these data suggest that rosiglitazone protects neurons from apoptosis in the brain tissues damaged by I/R.

To identify the effector protein that mediates the anti-apoptotic action of PPAR-*γ* agonists, we analyzed brain tissues by proteomics [35]. Ischemic brain tissues from rats treated with or without rosiglitazone are collected and processed, and the lysate proteins from the tissues are analyzed by two-dimensional electrophoresis. A number of protein spots are enhanced in rosiglitazone-treated brain tissues. The spot that exhibits the highest increase (>5 fold) is removed and analyzed by tandem mass spectrometry. This protein spot matches 14-3-3*ε*. Western blot analysis of brain tissues confirms elevation of 14-3-3*ε* proteins in rosiglitazone-treated brain tissues. 14-3-3*ε* elevation in rosiglitazone-treated tissues is abrogated by concurrent administration of PPAR*γ* siRNA. Rosiglitazone-induced 14-3-3*ε* upregulation plays an important role in protecting against I/R-induced cerebral infarction. Silencing of brain 14-3-3*ε* with 14-3-3*ε* siRNA administration abrogates the anti-infarct effect of rosiglitazone while administration of 14-3-3*ε* attenuates I/R-induced infarction. Results from the *in vivo* experiments suggest that rosiglitazone at the concentrations used in our experiments protects brain tissues against I/R-induced damage via PPAR*γ*/14-3-3*ε*. It is unclear whether the negative effect of rosiglitazone at higher concentrations is related to 14-3-3*ε* suppression.

## 8. Rosiglitazone Enhances PPAR*γ* Binding to and Activation of 14-3-3*ε* Promoter

Ligand-activated PPAR*γ* exerts its biological actions by suppressing the expression of proinflammatory genes through NF-*κ*B-dependent transcriptional mechanism [39, 40]. It stimulates the expression of a small number of genes and little is known about its transcriptional mechanism. Our studies show that rosiglitazone induces PPAR*γ* binding to the PPREs of 14-3-3*ε* promoter/enhancer and activates 14-3-3*ε* transcription. In a neuronal cell model, rosiglitazone increases 14-3-3*ε* promoter activity and its effect is abrogated when the PPRE region is deleted from the 14-3-3*ε* promoter construct. Chromatin immunoprecipitation analysis reveals that rosiglitazone induces PPAR*γ* binding to the region harboring PPAR response elements. Corresponding to 14-3-3*ε* promoter activation, rosiglitazone increases 14-3-3*ε* protein expression which is abrogated by GW9662, a PPAR*γ* antagonist, and by PPAR*γ* siRNA.

Rosiglitazone-induced 14-3-3*ε* plays a crucial role in protecting neuronal cells from apoptosis induced by hypoxia and reoxygenation [35]. Knockdown of 14-3-3*ε* with 14-3-3*ε* siRNA abrogated the protective effect of rosiglitazone, while 14-3-3*ε* overexpression attenuates hypoxia-induced apoptosis. The protective effect of PPAR*γ* overexpression is also abrogated by 14-3-3*ε* siRNA. Taken together, the findings indicate that the PPAR*γ*-mediated 14-3-3*ε* upregulation represents an important mechanism by which PPAR*γ* ligands protect cells and tissues from I/R damage. 

Several reports have shown that rosiglitazone and other glitazones protect neuronal survival accompanied by increased Bcl-2 expression [41]. We have shown that rosiglitazone rescues Bcl-2 but not Bcl-XL in neurons from hypoxia/reoxygenation-induced repression [42]. As 14-3-3*ε* upregulation increases Bad sequestration, and, therefore, reduces Bad translocation to mitochondria to interfere with the protective action of Bcl-2, an enhanced Bcl-2 expression should further strengthen the protection of mitochondrial membrane potential and reduction of apoptosis.

## 9. PPAR*α* Ligands Protect against I/R Tissue Damage and Cell Death

PPAR*α* is activated by fatty acids, eicosanoids, and synthetic ligand such as fibrates, which are clinically used in treating dyslipidemia [43, 44]. In addition to their effects on glucose homeostasis and lipid metabolism [43], PPAR*α* ligands inhibit NF-*κ*B and AP-1 transactivation resulting in suppressing the expression of proinflammatory genes such as cyclooxygenase-2 (COX-2), inducible nitric oxide synthase (iNOS), and adhesive molecules ICAM-1 and VCAM-1 [45–48]. Ligand-activated PPAR*α* induces the expression of antioxidant enzymes including superoxide dismutase and catalase [49, 50]. Thus, ligand-activated PPAR*α* possesses anti-inflammatory and antioxidation properties.

Based on their potent anti-inflammatory and antioxidation actions, the synthetic PPAR*α* ligands, fibrates, have been used to control I/R-induced tissue injury. Chronic fenofibrate administration was shown to reduce infarct volume in a mouse middle cerebral artery occlusion model [51]. PGI_2_ overproduction via Ad-COPI gene transfer was shown to reduce renal I/R injury through PPAR*α* nuclear translocation [52]. WY14643 was reported to ameliorate cisplatin-induced renal damage [53]. Although the protective effects of PPAR*α* ligands on diverse I/R-induced tissue injuries are attributed to control of inflammatory and oxidative tissue damage, a number of reports indicate that ligand-activated PPAR*α* protects against apoptosis. For example, it was reported that PGI_2_ or docosahexaenoic acid protects renal cells from toxin-induced apoptosis [54, 55]; fenofibrate inhibits aldosterone-induced myocardiocyte apoptosis [56] and WY14643 prevents neonatal cardiomyocyte apoptosis induced by glucose and fatty acids [57]. The anti-apoptotic actions of ligand-activated PPAR*α* are likely to make significant contributions to protect tissues from I/R damage.

The mechanism by which PPAR*α* protects against apoptosis remains to be elucidated. We postulate that ligand-activated PPAR*α* confers anti-apoptotic protection also through binding to 14-3-3*ε* promoter and upregulating 14-3-3*ε* expression. The rationale for the proposed hypothesis is based on (1) high-sequence homology and structural similarity of PPAR*α* DNA binding domain with its counterparts in PPAR*γ* and PPAR*δ*, (2) identical cis-regulatory element motif that is recognized by PPAR*α*, PPAR*γ*, and PPAR*δ*, and (3) requirement of identical heterodimer partner, RXR for DNA binding. Work is in progress to test this hypothesis.

## 10. PPARs Defend against Mitochondrial Death Pathway by a Coordinated Common Mechanism

**Figure 1:**
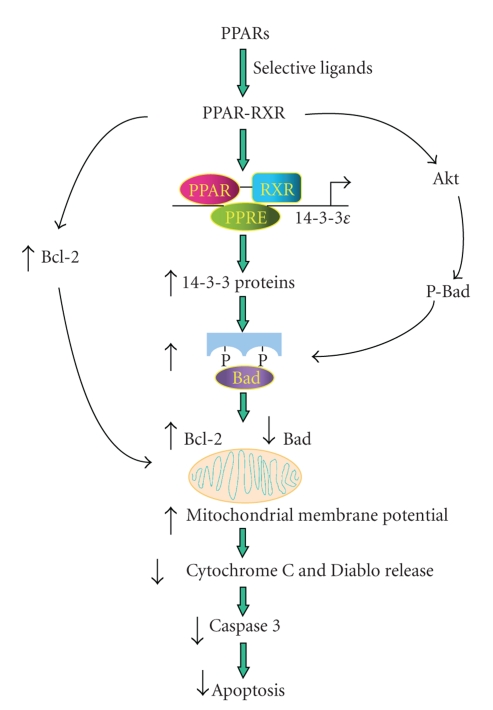
Schematic illustration of proposed signaling pathway by which all three PPAR isoforms exert anti-apoptotic actions via 14-3-3*ε* upregulation.

Based on findings reported by several laboratories including ours, we propose a common mechanism by which all three PPAR isoforms protect cells from oxidative mitochondrial damage and thereby defend against apoptosis via the intrinsic death pathway. As illustrated in Figure 1, PPAR*α*, *γ*, or *δ* activated by their respective ligands forms heterodimers with RXR which binds to the PPRE sites on the 14-3-3*ε* promoter and upregulates the transcription of 14-3-3*ε*. Enhanced 14-3-3*ε* augments binding and sequestration of Bad, and thereby reduces interference of Bcl-2 and Bcl-xl protective actions by Bad [58–61]. Mitochondrial membrane potential is maintained, and release of pro-apoptotic cofactors such as cytochrome C and Diablo is blocked when cells are challenged by oxidative stress and cytotoxic insults [58]. This results in reduction of caspase activation and caspase-induced apoptotic changes. 

Reported data indicate that ligand-activated PPAR*γ* activates Akt which phosphorylates Bad and enhances Bad binding by 14-3-3 [15, 33, 62]. Furthermore, ligand-activated PPAR*γ* stimulates Bcl-2 generation which enforces the mitochondrial protection [21, 42]. It is unclear whether ligand-activated PPAR*α* and PPAR*δ* have similar actions as PPAR*γ* on Akt activation and/or Bcl-2 upregulation.

## 11. Therapeutic Implications

 Ischemia-reperfusion tissue damage is one of the most important pathophysiological processes that cause major human diseases such as myocardial infarction (MI), stroke, and kidney diseases. Since PPAR*α* and PPAR*δ* ligands are unequivocally effective in preventing and interrupting I/R-induced infarction in experimental animals, they have potentials for therapeutic use in early treatment of MI, renal diseases, and stroke. Some of the synthetic agonists of PPAR*α* (the fibrates) and PPAR*δ* (PGI_2_ analogs) are already in use clinically for treating lipid and vascular disorders, respectively, and new compounds are undergoing clinical trials. Those drugs should be good candidates for therapy of MI, stroke, and other tissue infarctions. PPAR*α* and PPAR*δ* may be used individually or in combination. Some compounds such as carbaprostacyclin bind and activate PPAR*δ* and PPAR*α* and are well suited for therapeutic purposes.

The effects of PPAR*γ* agonists on controlling I/R damage are complex and dose-dependent because of their pleiotropic actions, some of which are independent of PPAR*γ*-transcriptional activities. Hence, despite beneficial effects reported by a majority of studies, PPAR*γ* agonists may be associated with adverse effects. Further studies are needed to unravel the mechanisms by which PPAR*γ* agonists exert a biphasic effects on cytoprotection.

PPARs/14-3-3*ε* axis may serve as targets for new drug discovery. Compounds that selectively activate this transcriptional pathway will be more specific and more potent in cell and tissue protection and possess less adverse effects.

## References

[1] Robinson E, Grieve DJ (2009). Significance of peroxisome proliferator-activated receptors in the cardiovascular system in health and disease. *Pharmacology and Therapeutics*.

[2] Bishop-Bailey D, Bystrom J (2009). Emerging roles of peroxisome proliferator-activated receptor-*β*/*δ* in inflammation. *Pharmacology and Therapeutics*.

[3] Kersten S, Desvergne B, Wahli W (2000). Roles of PPARS in health and disease. *Nature*.

[4] Liou J-Y, Lee S, Ghelani D, Matijevic-Aleksic N, Wu KK (2006). Protection of endothelial survival by peroxisome proliferator-activated receptor-*δ* mediated 14-3-3 upregulation. *Arteriosclerosis, Thrombosis, and Vascular Biology*.

[5] Wu KK, Liou J-Y (2005). Cellular and molecular biology of prostacyclin synthase. *Biochemical and Biophysical Research Communications*.

[6] Pober JS, Cotran RS (1990). Cytokines and endothelial cell biology. *Physiological Reviews*.

[7] Bunting S, Moncada S, Vane JR (1983). The prostacyclin-thromboxane A2 balance: pathophysiological and therapeutic implications. *British Medical Bulletin*.

[8] Oates JA, FitzGerald GA, Branch RA, Jackson EK, Knapp HR, Roberts LJ (1988). Clinical implications of prostaglandin and thromboxane A2 formation (first of two parts). *New England Journal of Medicine*.

[9] Hata AN, Breyer RM (2004). Pharmacology and signaling of prostaglandin receptors: multiple roles in inflammation and immune modulation. *Pharmacology and Therapeutics*.

[10] Adderley SR, Fitzgerald DJ (1999). Oxidative damage of cardiomyocytes is limited by extracellular regulated kinases 1/2-mediate induction of cyclooxygenase-2. *Journal of Biological Chemistry*.

[11] Hao C-M, Kömhoff M, Guan Y, Redha R, Breyer MD (1999). Selective targeting of cyclooxygenase-2 reveals its role in renal medullary interstitial cell survival. *American Journal of Physiology—Renal Physiology*.

[12] Pozner RG, Negrotto S, D’Atri LP (2005). Prostacyclin prevents nitric oxide-induced megakaryocyte apoptosis. *British Journal of Pharmacology*.

[13] Forman BM, Chen J, Evans RM (1997). Hypolipidemic drugs, polyunsaturated fatty acids, and eicosanoids are ligands for peroxisome proliferator-activated receptors *α* and *δ*. *Proceedings of the National Academy of Sciences of the United States of America*.

[14] Hao C-M, Redha R, Morrow J, Breyer MD (2002). Peroxisome proliferator-activated receptor *δ* activation promotes cell survival following hypertonic stress. *Journal of Biological Chemistry*.

[15] Di-Po N, Tan NS, Michalik L, Wahli W, Desvergne B (2002). Antiapoptotic role of PPAR*β* in keratinocytes via transcriptional control of the Akt1 signaling pathway. *Molecular Cell*.

[16] Pesant M, Sueur S, Dutartre P (2006). Peroxisome proliferator-activated receptor *δ* (PPAR*δ*) activation protects H9c2 cardiomyoblasts from oxidative stress-induced apoptosis. *Cardiovascular Research*.

[17] Wan J, Jiang L, Lü Q, Ke L, Li X, Tong N (2010). Activation of PPAR*δ* up-regulates fatty acid oxidation and energy uncoupling genes of mitochondria and reduces palmitate-induced apoptosis in pancreatic *β*-cells. *Biochemical and Biophysical Research Communications*.

[18] Kim HJ, Kim MY, Jin H (2009). Peroxisome proliferator-activated receptor *δ* regulates extracellular matrix and apoptosis of vascular smooth muscle cells through the activation of transforming growth factor-*β*1/Smad3. *Circulation Research*.

[19] Lin H, Lin T-N, Cheung W-M (2002). Cyclooxygenase-1 and bicistronic cyclooxygenase-1/ prostacyclin synthase gene transfer protect against ischemic cerebral infarction. *Circulation*.

[20] Fu H, Subramanian RR, Masters SC (2000). 14-3-3 Proteins: structure, function, and regulation. *Annual Review of Pharmacology and Toxicology*.

[21] Morrison DK (2009). The 14-3-3 proteins: integrators of diverse signaling cues that impact cell fate and cancer development. *Trends in Cell Biology*.

[22] Liou J-Y, Matijevic-Aleksic N, Lee S, Wu KK (2007). Prostacyclin inhibits endothelial cell XIAP ubiquitination and degradation. *Journal of Cellular Physiology*.

[23] Shiff SJ, Qiao L, Tsai L-L, Rigas B (1995). Sulindac sulfide, an aspirin-like compound, inhibits proliferation, causes cell cycle quiescence, and induces apoptosis in HT-29 colon adenocarcinoma cells. *Journal of Clinical Investigation*.

[24] DuBois RN, Abramson SB, Crofford L (1998). Cyclooxygenase in biology and disease. *FASEB Journal*.

[25] Piazza GA, Alberts DS, Hixson LJ (1997). Sulindac sulfone inhibits azoxymethane-induced colon carcinogenesis in rats without reducing prostaglandin levels. *Cancer Research*.

[26] Gupta RA, Tan J, Krause WF (2000). Prostacyclin-mediated activation of peroxisome proliferator-activated receptor *δ* in colorectal cancer. *Proceedings of the National Academy of Sciences of the United States of America*.

[27] Gupta RA, Wang D, Katkuri S, Wang H, Dey SK, DuBois RN (2004). Activation of nuclear hormone receptor peroxisome proliferator-activated receptor-*δ* accelerates intestinal adenoma growth. *Nature Medicine*.

[28] He T-C, Chan TA, Vogelstein B, Kinzler KW (1999). PPAR*δ* is an APC-regulated target of nonsteroidal anti-inflammatory drugs. *Cell*.

[29] Liou J-Y, Ghelani D, Yeh S, Wu KK (2007). Nonsteroidal anti-inflammatory drugs induce colorectal cancer cell apoptosis by suppressing 14-3-3*ε*. *Cancer Research*.

[30] Liou J-Y, Wu C-C, Chen B-R, Yen LB, Wu KK (2008). Nonsteroidal anti-inflammatory drugs induced endothelial apoptosis by perturbing peroxisome proliferator-activated receptor-*δ* transcriptional pathway. *Molecular Pharmacology*.

[31] Ho T-C, Chen S-L, Yang Y-C, Liao C-L, Cheng H-C, Tsao Y-P (2007). PEDF induces p53-mediated apoptosis through PPAR gamma signaling in human umbilical vein endothelial cells. *Cardiovascular Research*.

[32] Okura T, Nakamura M, Takata Y, Watanabe S, Kitami Y, Hiwada K (2000). Troglitazone induces apoptosis via the p53 and Gadd45 pathway in vascular smooth muscle cells. *European Journal of Pharmacology*.

[33] Kilter H, Werner M, Roggia C (2009). The PPAR-*γ* agonist rosiglitazone facilitates Akt rephosphorylation and inhibits apoptosis in cardiomyocytes during hypoxia/reoxygenation. *Diabetes, Obesity and Metabolism*.

[34] Lin C-Y, Gurlo T, Haataja L, Hsueh WA, Butler PC (2005). Activation of peroxisome proliferator-activated receptor-*γ* by rosiglitazone protects human islet cells against human islet amyloid polypeptide toxicity by a phosphatidylinositol 3′-kinase-dependent pathway. *Journal of Clinical Endocrinology and Metabolism*.

[35] Wu J-S, Cheung W-M, Tsai Y-S (2009). Ligand-activated peroxisome proliferator-activated receptor-*γ* protects against ischemic cerebral infarction and neuronal apoptosis by 14-3-3*ε* upregulation. *Circulation*.

[36] Hinz B, Brune K, Pahl A (2003). 15-Deoxy- Δ12,14-prostaglandin J2 inhibits the expression of proinflammatory genes in human blood monocytes via a PPAR-*γ*-independent mechanism. *Biochemical and Biophysical Research Communications*.

[37] Lynn Wang Y, Frauwirth KA, Rangwala SM, Lazar MA, Thompson CB (2002). Thiazolidinedione activation of peroxisome proliferator-activated receptor *γ* can enhance mitochondrial potential and promote cell survival. *Journal of Biological Chemistry*.

[38] Lin T-N, Cheung W-M, Wu J-S (2006). 15d-prostaglandin J2 protects brain from ischemia-reperfusion injury. *Arteriosclerosis, Thrombosis, and Vascular Biology*.

[39] Ricote M, Li AC, Willson TM, Kelly CJ, Glass CK (1998). The peroxisome proliferator-activated receptor-*γ* is a negative regulator of macrophage activation. *Nature*.

[40] Jiang C, Ting AT, Seed B (1998). PPAR-*γ* agonists inhibit production of monocyte inflammatory cytokines. *Nature*.

[41] Fuenzalida K, Quintanilla R, Ramos P (2007). Peroxisome proliferator-activated receptor *γ* up-regulates the Bcl-2 anti-apoptotic protein in neurons and induces mitochondrial stabilization and protection against oxidative stress and apoptosis. *Journal of Biological Chemistry*.

[42] Wu J-S, Lin T-N, Wu KK (2009). Rosiglitazone and PPAR-*γ* overexpression protect mitochondrial membrane potential and prevent apoptosis by upregulating anti-apoptotic Bcl-2 family proteins. *Journal of Cellular Physiology*.

[43] Barbier O, Torra IP, Duguay Y (2002). Pleiotropic actions of peroxisome proliferator-activated receptors in lipid metabolism and atherosclerosis. *Arteriosclerosis, Thrombosis, and Vascular Biology*.

[44] Lefebvre P, Chinetti G, Fruchart J-C, Staels B (2006). Sorting out the roles of PPAR*α* in energy metabolism and vascular homeostasis. *Journal of Clinical Investigation*.

[45] Delerive P, De Bosscher K, Besnard S (1999). Peroxisome proliferator-activated receptor *α* negatively regulates the vascular inflammatory gene response by negative cross-talk with transcription factors NF-*κ*B and AP-1. *Journal of Biological Chemistry*.

[46] Staels B, Koenig W, Habib A (1998). Activation of human aortic smooth-muscle cells is inhibited by PPAR*α* but not by PPAR*γ* activators. *Nature*.

[47] Marx N, Sukhova GK, Collins T, Libby P, Plutzky J (1999). PPAR*α* activators inhibit cytokine-induced vascular cell adhesion molecule-1 expression in human endothelial cells. *Circulation*.

[48] Wayman NS, Hattori Y, Mcdonald MC (2002). Ligands of the peroxisome proliferator-activated receptors (PPAR-*γ* and PPAR-*α*) reduce myocardial infarct size. *FASEB Journal*.

[49] Inoue I, Goto S-I, Matsunaga T (2001). The ligands/activators for peroxisome proliferator-activated receptor *α* (PPAR*α*) and PPAR*γ* increase Cu^2+^,Zn^2+^-superoxide dismutase and decrease p22phox message expressions in primary endothelial cells. *Metabolism*.

[50] Poynter ME, Daynes RA (1998). Peroxisome proliferator-activated receptor *α* activation modulates cellular redox status, represses nuclear factor-*κ*B signaling, and reduces inflammatory cytokine production in aging. *Journal of Biological Chemistry*.

[51] Deplanque D, Gelé P, Pétrault O (2003). Peroxisome proliferator-activated receptor-*α* activation as a mechanism of preventive neuroprotection induced by chronic fenofibrate treatment. *Journal of Neuroscience*.

[52] Chen H-H, Chen T-W, Lin H (2009). Prostacyclin-induced peroxisome proliferator-activated receptor-*α* translocation attenuates NF-*κ*B and TNF-*α* activation after renal ischemia-reperfusion injury. *American Journal of Physiology—Renal Physiology*.

[53] Li S, Bhatt R, Megyesi J, Gokden N, Shah SV, Portilla D (2004). PPAR-*α* ligand ameliorates acute renal failure by reducing cisplatin-induced increased expression of renal endonuclease G. *American Journal of Physiology—Renal Physiology*.

[54] Lin H, Hou C-C, Cheng C-F (2007). Peroxisomal proliferator-activated receptor-*α* protects renal tubular cells from doxorubicin-induced apoptosis. *Molecular Pharmacology*.

[55] Chen H-H, Sue Y-M, Chen C-H (2009). Peroxisome proliferator-activated receptor alpha plays a crucial role in l-carnitine anti-apoptosis effect in renal tubular cells. *Nephrology Dialysis Transplantation*.

[56] De Silva DS, Wilson RM, Hutchinson C (2009). Fenofibrate inhibits aldosterone-induced apoptosis in adult rat ventricular myocytes via stress-activated kinase-dependent mechanisms. *American Journal of Physiology—Heart and Circulatory Physiology*.

[57] Wu QN, Tang Q, Xiao Q, Wu P, Gao A, Li L PPARalpha agonist prevented the apoptosis induced by glucose and fatty acid in neonatal cardiomyocytes.

[58] Hengartner MO (2000). The biochemistry of apoptosis. *Nature*.

[59] Datta SR, Katsov A, Hu L (2000). 14-3-3 proteins and survival kinases cooperate to inactivate BAD by BH3 domain phosphorylation. *Molecular Cell*.

[60] Zha J, Harada H, Yang E, Jockel J, Korsmeyer SJ (1996). Serine phosphorylation of death agonist BAD in response to survival factor results in binding to 14-3-3 not BCL-X(L). *Cell*.

[61] Nomura M, Shimizu S, Sugiyama T (2003). 14-3-3 Interacts directly with and negatively regulates pro-apoptotic Bax. *Journal of Biological Chemistry*.

[62] Han JK, Lee HS, Yang HM (2008). Peroxisome proliferator-activated receptor-*δ* agonist enhances vasculogenesis by regulating endothelial progenitor cells through genomic and nongenomic activations of the phosphatidylinositol 3-kinase/Akt pathway. *Circulation*.

